# Supplementary material for: Re-Evaluation of Genotyping Methodologies in Cattle: The Proficiency of Imputation
Source: Genes (Basel). 2023 Feb 22;14(3):547. doi: 10.3390/genes14030547 (PMC10048120; doi:10.3390/genes14030547)
Supplement: Supplementary file 1 [file genes-14-00547-s001.zip › genes-2127485-supplementary.pdf]

File S1: *BTA14 association analysis of RFI*

To search for RFI QTLs, we used the BovineHD genotyping data of BTA14 of the studied 192 cows, whereas the response variable was RFI. The files were prepared and formatted as described in [1] using the Plink software [2]. The nominal probabilities for the hypothesis of no effect and the substitution effects were computed using EMMAX software [3] as previously described [4]. Briefly, a relationship matrix was generated to account for the relationships across cows, based on the identity-by-state matrix calculated using the emmax-kin-intel64 algorithm with the -v -s -d 10 flags. The association was computed by the EMMAX algorithm with the -v -d 10 –t flags, and the -k argument was used for including the relationship matrix. This analysis pointed to one QTL around BTA14 position 16.79 Kb peaking at SNP BovineHD1400005275.

References

1. Gershoni, M.; Ezra, E.; Weller, J.I. Genetic and genomic analysis of long insemination interval in Israeli dairy cattle as an indicator of early abortions. J Dairy Sci 2020, 103, 4495-4509, https://doi.org/10.3168/jds.2019-17482.

2. Purcell, S.; Neale, B.; Todd-Brown, K.; Thomas, L.; Ferreira, M.A.R.; Bender, D.; Maller, J.; Sklar, P.; de Bakker, P.I.W.; Daly, M.J., et al. PLINK: A tool set for whole-genome association and population-based linkage analyses. Am J Hum Genet 2007, 81, 559-575, https://doi.org/10.1086/519795.

3. Kang, H.M.; Sul, J.H.; Service, S.K.; Zaitlen, N.A.; Kong, S.Y.; Freimer, N.B.; Sabatti, C.; Eskin, E. Variance component model to account for sample structure in genome-wide association studies. Nat Genet 2010, 42, 348-354. https://doi.org/10.1038/ng.548.

4. Weller, J.I.; Ezra, E.; Gershoni, M. Genetic and genomic analysis of age at first insemination in Israeli dairy cattle. Journal of Dairy Science 2022, 105, 5192-5205, https://doi.org/10.3168/jds.2021-21528.

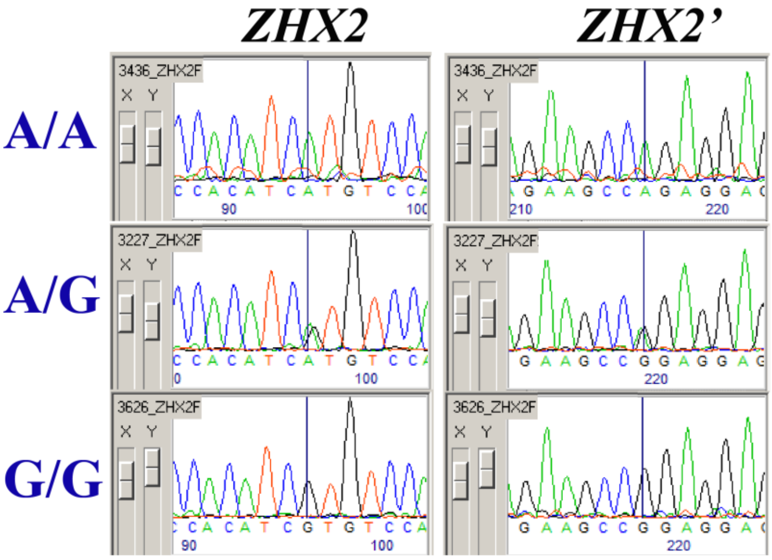

Figure S1. *ZHX2* polymorphism. Trace chromatogram obtained from three cows (3436, 3227 and 3626) were chosen to corroborate imputed genotypes and to illustrate typical results at two SNP sites within exon 1, for the three genotype states (A/A, A/G and G/G, blue font). The presented sequence traces were derived from three chromatogram files for which the *ZHX2* variation (on the left, within trace positions 90-100) was tightly linked to the *ZHX2'* variation (on the right, within trace positions 210-220), helping to corroborate the three genotype states (blue font).

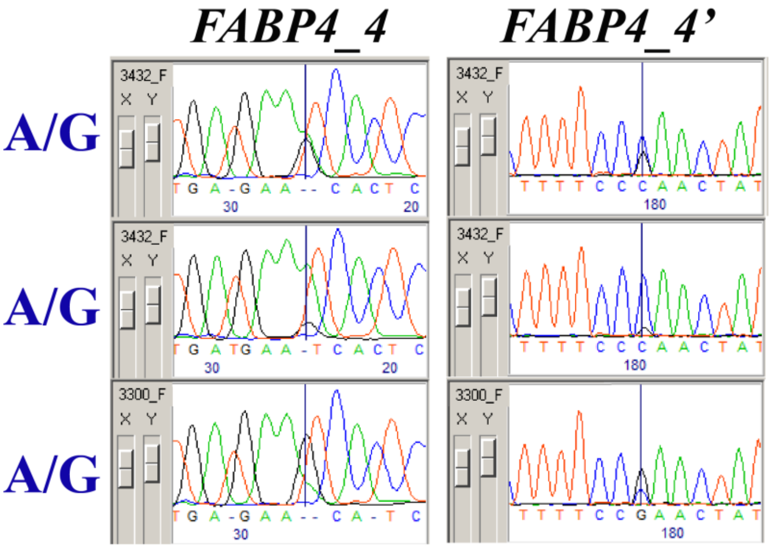

Figure S2. Imbalanced allele proportions in Sanger sequencing. Trace chromatogram obtained from three cows (3432, 3300) demonstrated random distortions in the observed allele proportions in the *FABP4_4* traces. All chromatograms represent the heterozygous A/G state.

Table S1. PCR primers.

Marker	Forward	Reverse	Sequencing direction	Sequencing primer
FABP4_4	CCTTTCCCCCGACTCTTTTA	TTGTGCCTTGGGTGTTCTTT	Forward	TCTGCTCCAGTTCTTTATTTCT
FABP4_5	AAGTGCAGCTTTCAGCAAGA	TACCCCACTCCAGATTGCAT	Reverse	TACCCCACTCCAGATTGCAT
ZHX2	CGACTCCTTGTCTGACCACA	GAGGATGGGCTGTGACATCT	Reverse	GAGGATGGGCTGTGACATCT
